# Identification of *R2R3-MYB* family in blueberry and its potential involvement of anthocyanin biosynthesis in fruits

**DOI:** 10.1186/s12864-023-09605-w

**Published:** 2023-08-30

**Authors:** Haiyang Wang, Lulu Zhai, Shouwen Wang, Botian Zheng, Honglu Hu, Xuyan Li, Shaomin Bian

**Affiliations:** College of Plant Science, Jilin University, Changchun, China

**Keywords:** *Vaccinium corymbosum*, *R2R3-MYBs*, Anthocyanin biosynthesis, Gene expression

## Abstract

**Background:**

Blueberries (*Vaccinium corymbosum*) are regarded as “superfoods” attributed to large amounts of anthocyanins, a group of flavonoid metabolites, which provide pigmentation in plant and beneficial effects for human health. MYB transcription factor is one of vital components in the regulation of plant secondary metabolism, which occupies a dominant position in the regulatory network of anthocyanin biosynthesis. However, the role of MYB family in blueberry responding to anthocyanin biosynthesis remains elusive.

**Results:**

In this study, we conducted a comprehensive analysis of *VcMYB*s in blueberry based on the genome data, including phylogenetic relationship, conserved motifs, identification of differentially expressed *MYB* genes during fruit development and their expression profiling, etc. A total of 437 unique MYB sequences with two SANT domains were identified in blueberry, which were divided into 3 phylogenetic trees. Noticeably, there are many trigenic and tetragenic *VcMYB*s pairs with more than 95% identity to each other. Meanwhile, the transcript accumulations of *VcMYB*s were surveyed underlying blueberry fruit development, and they showed diverse expression patterns, suggesting various functional roles in fruit ripening. More importantly, distinct transcript profiles between skin and pulp of ripe fruit were observed for several *VcMYBs*, such as *VcMYB437*, implying the potential roles in anthocyanin biosynthesis.

**Conclusions:**

Totally, 437 VcMYBs were identified and characterized. Subsequently, their transcriptional patterns were explored during fruit development and fruit tissues (skin and pulp) closely related to anthocyanin biosynthesis. These genome-wide data and findings will contribute to demonstrating the functional roles of VcMYBs and their regulatory mechanisms for anthocyanins production and accumulation in blueberry in the future study.

**Supplementary Information:**

The online version contains supplementary material available at 10.1186/s12864-023-09605-w.

## Background

Blueberries (*Vaccinium* spp.), a member of the family Ericaceae, are perennial flowering shrubs with indigo-colored small berries, as an addition, they also known as lingonberries [1]. Its unique flavor, rich nutrients are the mainly reasons that people love this economically important crop, moreover, it can exactly prevent multiple diseases, such as neurodegenerative disease, cardiovascular disease and cancer in epidemiological research [2]. Noticeably, these quality traits which are good for healthy of human are largely determined by the anthocyanins keeping a high level in many berries, which is also the important role generating the colorful pigments in many flowers and fruits, for examples, bilberry (*Vaccinium myrtillus*) and strawberry (*Fragaria* spp.) all are colored by anthocyanins [3]. Diverse plant species in landscape gardens keep a high level of anthocyanins in their different tissues, such as grape (*Vitis vinifera*), eggplant (*Solanum melongena*), Chinese cabbage (*Brassica rapa* L.), parsley (*Petroselinum crispum*), apple (*Malus domestica*), strawberry (*Fragaria vesca*), mulberry (*Morus alba*), and petunia (*Petunia hybrida*) containing a large number of anthocyanins in leaves, stems, roots, flowers and fruits [4–9]. A category of flavonoids in plant, anthocyanins contribute to the blueberry’s color [10, 11], as well as its antioxidant properties [12]. In view of this, a great deal of research has been done on the regulation of anthocyanin synthesis as a result. In a number of plant species, it has been well demonstrated that genetic factors influence the biosynthesis of anthocyanins [13, 14]. Diverse transcription factors (TF) like MYB, basic helix-loop-helix (bHLH), WD40 and WRKY can mainly control the expression of structural genes encoding enzymes involved in the anthocyanin biosynthesis pathway at the transcriptional level [15], in which the MBW (MYB-bHLH-WD40) transcription complex is crucial, especially.

As previous studies, MYB is one of the largest TF families, consisting of three helix-turn-helix structures of approximately 53 amino acids per conserved repeat [16]. Four subfamilies (1R-, 3R-, 4R- and R2R3-MYB, respectively) composed of the whole MYB proteins family, which was classified according to the number and identity of the repeats in the MYB domain [17, 18]. It has been proved that R2R3-MYB TFs are very specific in plant for they can just be detective in plants and play important roles involved the plant specific processes including defense, root genesis and development [19]. Nevertheless, for most R2R3-type *MYB* genes on blueberries, fewer functional molecular data are available compared to other species.

It has been shown that anthocyanin biosynthesis in many plants is determined by R2R3-type MYBs, such as *Arabidopsis thaliana* [20], grape (*Vitis vinifera*) [21], apple (*Malus domestica*) [22], strawberry (*Fragaria vesca*) [23], pear (*Pyrus* L.) [24], tomato (*Solanum lycopersicum*) [25], Chinese cabbage (*Brassica rapa* L.) [26] and Purple tea [*Camellia sinensis* var. *asssamica* (Masters) kitamura] [27, 28]. Previous studies have identified numerous activators for flavonoid and anthocyanin biosynthesis. In apple, MdMYB1 enhances the expression of *MdANS* (*anthocyanin synthase*), *MdDFR* (*dihydroflavonol 4 reductase*), and *MdUF3GT* (*UDP-glucose: flavonoid 3-o-glucosyltransferase*) by binding to their promoters directly and thereby elevates the anthocyanin level [22, 29, 30]. Anthocyanin pigment accumulation occurs in *Arabidopsis thaliana* when *AtMYB75/PAP1* (*production of anthocyanin pigment1*) and *AtMYB90/PAP2* are overexpressed [21]. After interacting with PyWRKY26, PybHLH3 binds to the promoter of *PyMYB114* and activates *PyMYB114* transcription, which therefore increases anthocyanin accumulation in red skinned pear [24]. In tomato, the expression of SlAN2-like, an R2R3-MYB, is regulated by SlAN1. When SlAN2-like and SlAN1 are knocked out, pigmentation of anthocyanins is abolished [25].

Recently, growing evidence reveals that R2R3-MYBs may also repress the synthesis of proanthocyanin and anthocyanin [4, 30–36]. AtMYB4, homolog of AtMYB7 and AtMYB32 [37], is the key transcription factor that can repress flavonol biosynthesis in *Arabidopsis thaliana*. It represses the early phenylpropanoid genes including the flavonoid-specific genes, *flavonoid 3’-hydroxylase* (*F3’H*), *DFR*, *phenylalanine ammonia-lyase* (*PAL*), *cinnamate 4-hydroxylase* (*C4H*) and *4-coumarate-CoA ligase* (*4CL*) [37–39]. The C2 repressor plays an important role in the SG4 subgroup, inhibiting a number of flavonoid pathways [40]. In grape (*Vitis vinifera*), VvMYB114 with repression motifs, inhibits the expression of *UFGT* (*UDP-glycose flavonoid glycosyltransferase*) and *DFR*, as a result of reduction the amount of anthocyanin biosynthesized [41]. It is, however, still not completely understood how these key players coordinate the various branches of flavonoid biosynthesis, especially in fruit, showing complicated flavonoids and anthocyanin profiles. For example, there is high accumulation of anthocyanins in the skin of blueberry, while only trace amounts are detected in the pulp. Therefore, it is concerned about whether there is a similar R2R3-MYB regulatory mechanism involved in anthocyanins biosynthesis in commercially important genus *Vaccinium* berries.

In previous studies of blueberries, anthocyanin accumulation is activated by MYBA-type TFs both in highbush blueberries and rabbiteye blueberries, while VcMYBPA1 may activate the biosynthesis of PA [42]. Anthocyanin biosynthesis is influenced by MYBPA1-type TFs in *Vaccinium* species, according to previous studies in *V. uliginosum*, *V. myrtillus* and in blueberry skin where *MYBPA1* expression is positively correlated with *ANS*, a key biosynthesis gene [42]. Moreover, *VcMYBA* and *VcMYB1* have been proved as important roles correlated with the expression of anthocyanin accumulation related structural gene, meanwhile they are able to transactivate the *DFR* promoter in blueberry fruit [1, 14]. Differently, MYBC2.1 and MYBR3.1, activated by MYBPA1, are demonstrated as two distinct MYB repressors of both PA and anthocyanin biosynthesis [43]. Nonetheless, in order to understand how the R2R3-MYB regulators are involved in anthocyanin biosynthesis, a comprehensive functional characterization is needed. As the present studies have proved, genome-wide investigation of the *MYB* genes in blueberry different fruit developments and tissues was accomplished. There are 437 unique *MYB* sequences were explored and classified into 3 phylogenetic trees. Totally of 347 and 138 *MYB* differentially expressed genes (DEGs) underlying various developmental stages and in different tissues were explored, respectively. Furthermore, important potential *VcMYB*s that might involve in the accumulation of anthocyanin were identified by analyzing expression patterns and identifying 2-fold DEGs. We examined the gene structures, motif architectures, chromosomal locations, and phylogenetic relationships of these genes. These results will be useful in addressing the potential functions of *VcMYB*s family in the future, especially for fruit development and anthocyanin accumulation.

## Results

### Characterization of *R2R3-MYB* family genes in blueberry

**Fig. 1:**
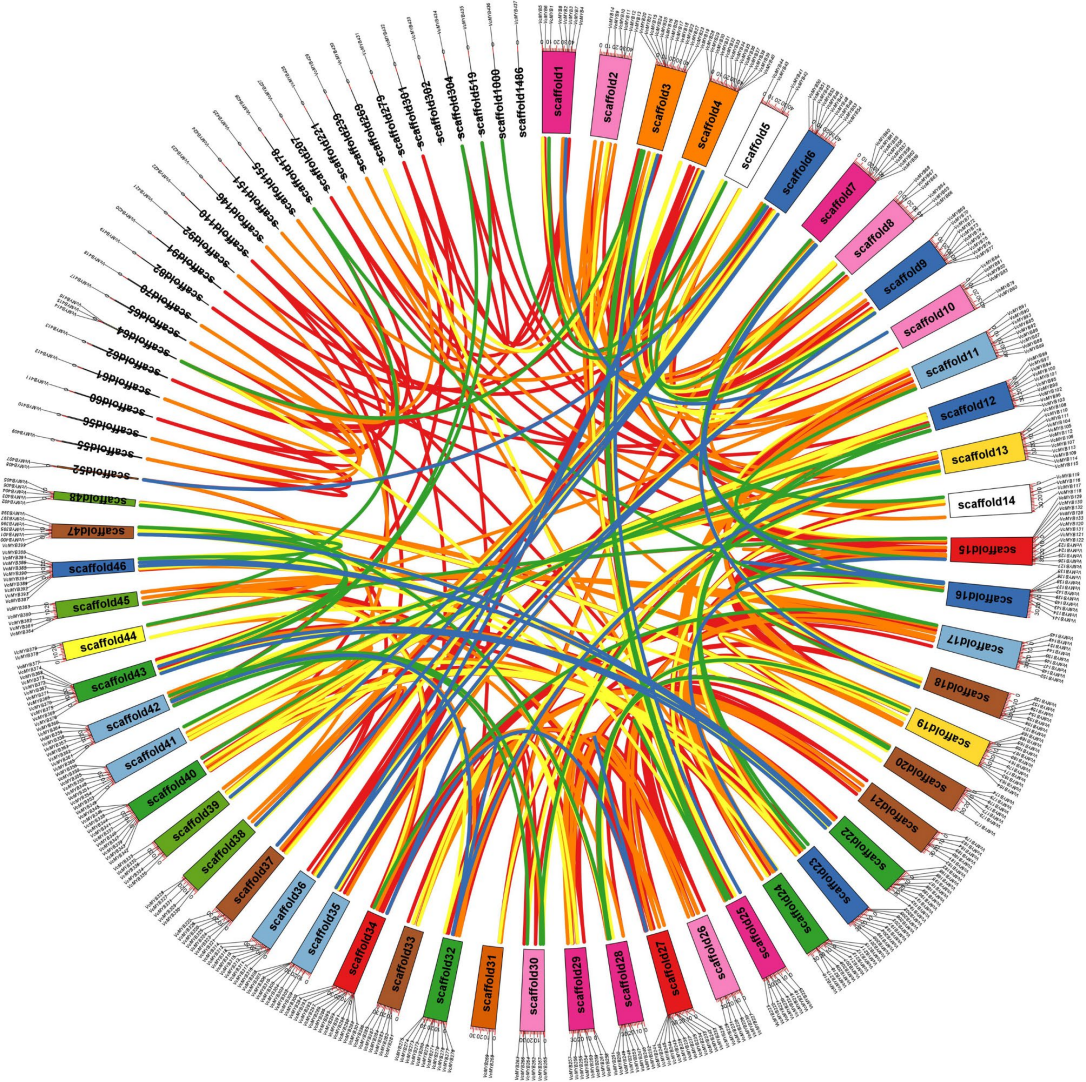
Chromosomal localization of the *VcMYB* family genes. Each box represents a scaffold, with different color meaning different number of *VcMYB* genes in one scaffold (red, orange, yellow, green, blue, light-blue, purple, brown, pink, yellow-green, white, light-yellow, light- brown and gray colors represent the number of *VcMYB* genes from 14 to 1, respectively). The approximate distribution of each *VcMYB* gene is marked on the circle with a short black line. Colored lines refer to the linkage group with high identity, red line 100%; orange line 99–100%; yellow line 98–99%; green 97–98%; brown 96–97%; blue 95–96%

After verification of R2R3-MYB family in blueberry by the hmmer model obtained from the Pfam website and two SANT domain on the SMART website, as well as removal of redundant sequences, 437 unique *R2R3-MYB* genes were totally identified and named as the number of scaffold (Fig. 1). In Table S1, the details of the information upon all the 437 *R2R3-MYB*s are listed. In brief, the range of *VcMYB* family CDS lengths, deduced proteins and the estimated molecular weights is 375–4995, 125–1665 and 14.26-181.28, respectively. And the range of protein isoelectric points is 4.79–10.45. These characteristics suggested the functional diversity for *VcMYB* family in blueberry.

### Chromosomal localization of *R2R3-MYB* family genes in blueberry and their evolutionary relationships

The corresponding scaffolds of the draft genome (1760 scaffolds) where these *R2R3-MYB* genes are localized was utilized to construct a Circos map, in order to investigate the distribution of *R2R3-MYB* family genes. There are 76 distinct scaffolds in which *R2R3-MYB*s are positioned unevenly. Twenty-six scaffolds harbor only one *R2R3-MYB* gene, such as scaffold 55, 56 and 1486, while two, three and four *R2R3-MYB* genes are separately distributed in each of two scaffolds (Fig. 1). Also, scaffold 15, 27, 34 harbor 14 *R2R3-MYB* genes, which is the most abundant *R2R3-MYB* genes in one scaffold. In addition to tandem and segmental duplications of chromosomal regions, whole-genome duplications (WGD) can also produce gene families [44–46]. Generally, tandem duplication makes a copy of a segment at genome level and subsequently inserts the one right after the segment with the distance no more than 200 kb [46]. It was observed that six pairs of *VcMYBs* (*VcMYB129* and *VcMYB130*, *VcMYB239* and *VcMYB244*, *VcMYB243* and *VcMYB244*, *VcMYB243* and *VcMYB245*, *VcMYB244* and *VcMYB245*, *VcMYB291* and *VcMYB299*) are separated by 14.8 kb, 139.9 kb, 82.8 kb, 129.1 kb, 48.5 kb and 58.6 kb, respectively (Fig. 1). Noticeably, *VcMYB239*, *VcMYB243*, *VcMYB244* and *VcMYB245* are all distributed at scaffold 27 and close to each other. The identities at nucleotide level of these six pair *VcMYB*s are all above 95% (Table S2-1), suggesting that tandem duplication might be responsible for the gene pairs. Summarizing the results of genetic analysis of *VcMYB* genes (Fig. 1), these *VcMYB* genes whose identity was above 95% potentially descended from a single common ancestor, and the duplication could result in a larger *VcMYB* gene family [46]. Furthermore, 53 digenic, 88 trigenic, and 243 tetragenic pairs of *VcMYB* (Fig. 1 and Table S2-1) exhibit quite high identity (over 95%), suggesting that they may derived from segmental or whole-genome duplication. Meanwhile, the ratio of non-synonymous/synonymous substitutions (Ka/Ks) was calculated between duplicated gene pairs. Except three gene pairs (*VcMYB239* and *VcMYB244*, *VcMYB243* and *VcMYB244*, *VcMYB243* and *VcMYB245*), all gene pairs have Ka/Ks values no more than 1 (Table S2-2), implying that these gene pairs and trigenic *VcMYB*s have probably gone through a purifying selection with limited diversified functions.

**Fig. 2:**
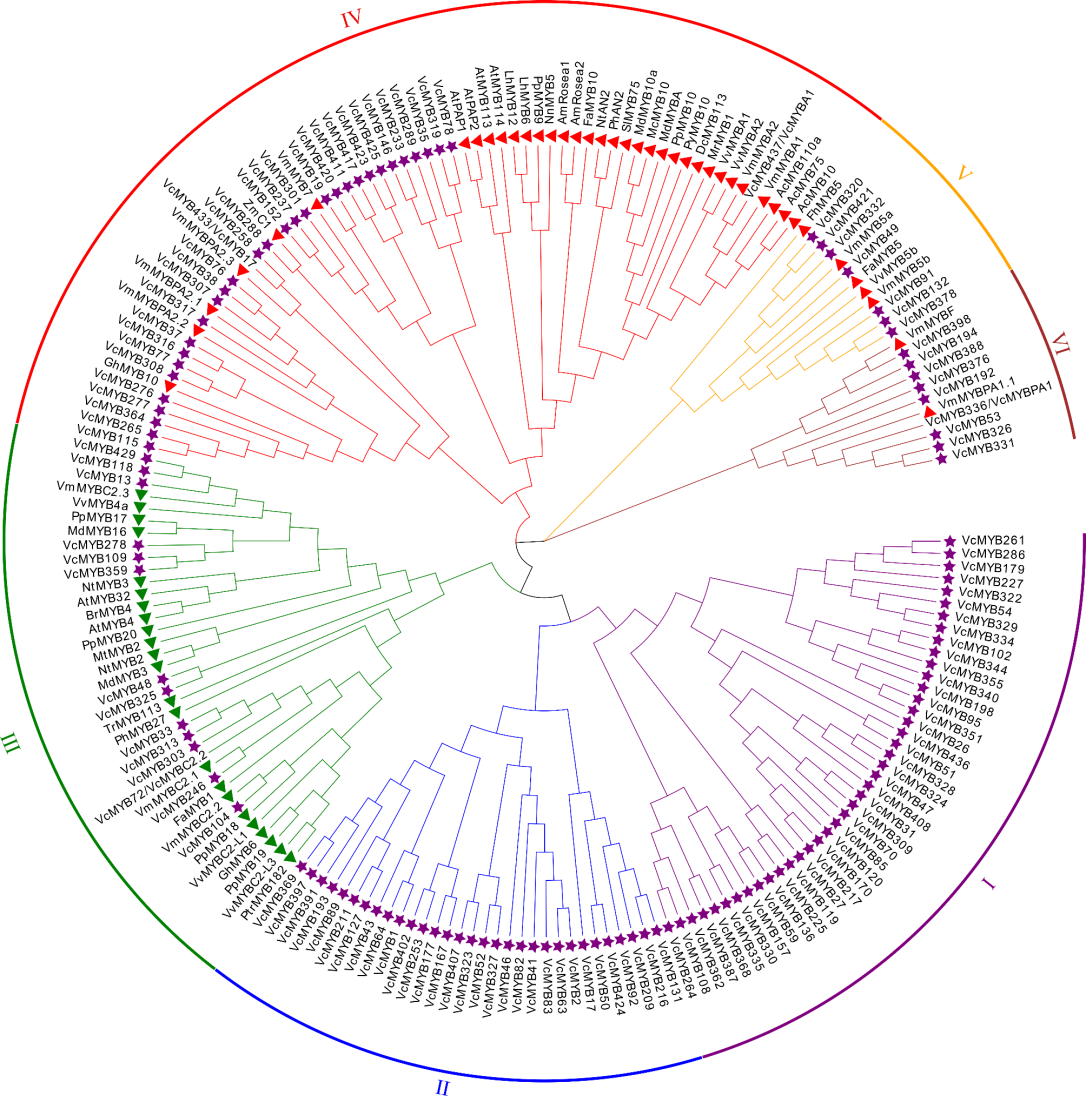
Phylogenetic relationship of VcMYB proteins with the MYBs in other plant species. The sequences of publicly known MYB proteins in bilberry, grape, apple, pear, petunia, peach, kiwi, snapdragon, carrot, fragrant sherry, strawberry, cotton, lily, bayberry, lotus, narcissus, tomato, Arabidopsis, poplar and corn were downloaded from NCBI database. 199 MYBs were showed in this phylogenetic tree1, and this phylogenetic tree was classified into 6 different groups and clustered together with MYBs from other plant species. All the R2R3-MYBs in other species that can act as activators of anthocyanin biosynthesis are represented with the same symbol: red triangle, and the ones that can act as repressors of anthocyanin biosynthesis are represented with the same symbol: green triangle. All the VcMYBs are represented with purple star. Four MYBs after slash means being already named previously

For elucidation their evolutionary relationship across species, phylogenetic analysis was conducted with the amino acid sequences of 503 R2R3-MYBs, including 66 MYBs from bilberry [47], grape, apple, pear, petunia, peach, kiwi, snapdragon, carrot, fragrant sherry, strawberry, cotton, lily, bayberry, lotus, narcissus, tomato, Arabidopsis, poplar and corn that worked as regulators in the biosynthesis of anthocyanin [48]. Consequently, based on the ML value, all the MYBs were divided into 3 phylogenetic trees, tree1 (Fig. 2), tree2 (Fig. S1A) and tree3 (Fig. S1B), which were composed of 199, 174 and 130 R2R3-MYBs, and classified into 6, 6 and 5 different subgroups, respectively. We selected the MYBs with the value between 504 ~ 701, 702 ~ 875 and 876 ~ 1004 to construct the phylogenetic tree 1, 2 and 3, respectively. All the MYBs from other plant species, except AtMYB3 [49] (in phylogenetic tree 2) and MdMYB6 [50] (in phylogenetic tree 3), were clustered in phylogenetic tree1. The VcMYBs which were either divided into one phylogenetic tree or one branch with function-known MYBs from other plant species were probably to have undergone similar evolutionary diversification and might have analogous functions for regulating the biosynthesis of flavonoids. For example, VcMYB437 which was reported as VcMYBA1 activating the biosynthesis of anthocyanin was clustered with AtMYBPAP1 in the clade IV from phylogenetic tree 1 (Fig. 2), which is associated with BHLH12/MYC1 [47], EGL3, or GL3 [51], and promotes the synthesis of phenylpropanoid-derived compounds such as anthocyanins and proanthocyanidin in plants [52, 53]. Meanwhile, VcMYB433 named as VcMYB17 by previous studies showed high homologous with VmPA2.3 playing an important role in the biosynthesis of anthocyanins. Moreover, VcMYB13, VcMYB118 and VcMYB429 were grouped with VmMYBC2.3 in the clade III of phylogenetic tree 1, who expressed in tissues with high PA content and participated in inhibition mechanisms that controlled distinct flavonoid pathways [47] as well as VcMYB72 (VcMYBC2.2) which was documented as the repressors by the previous studies [54].

### Phylogenetic and conserved motifs analysis of VcMYBs

**Fig. 3:**
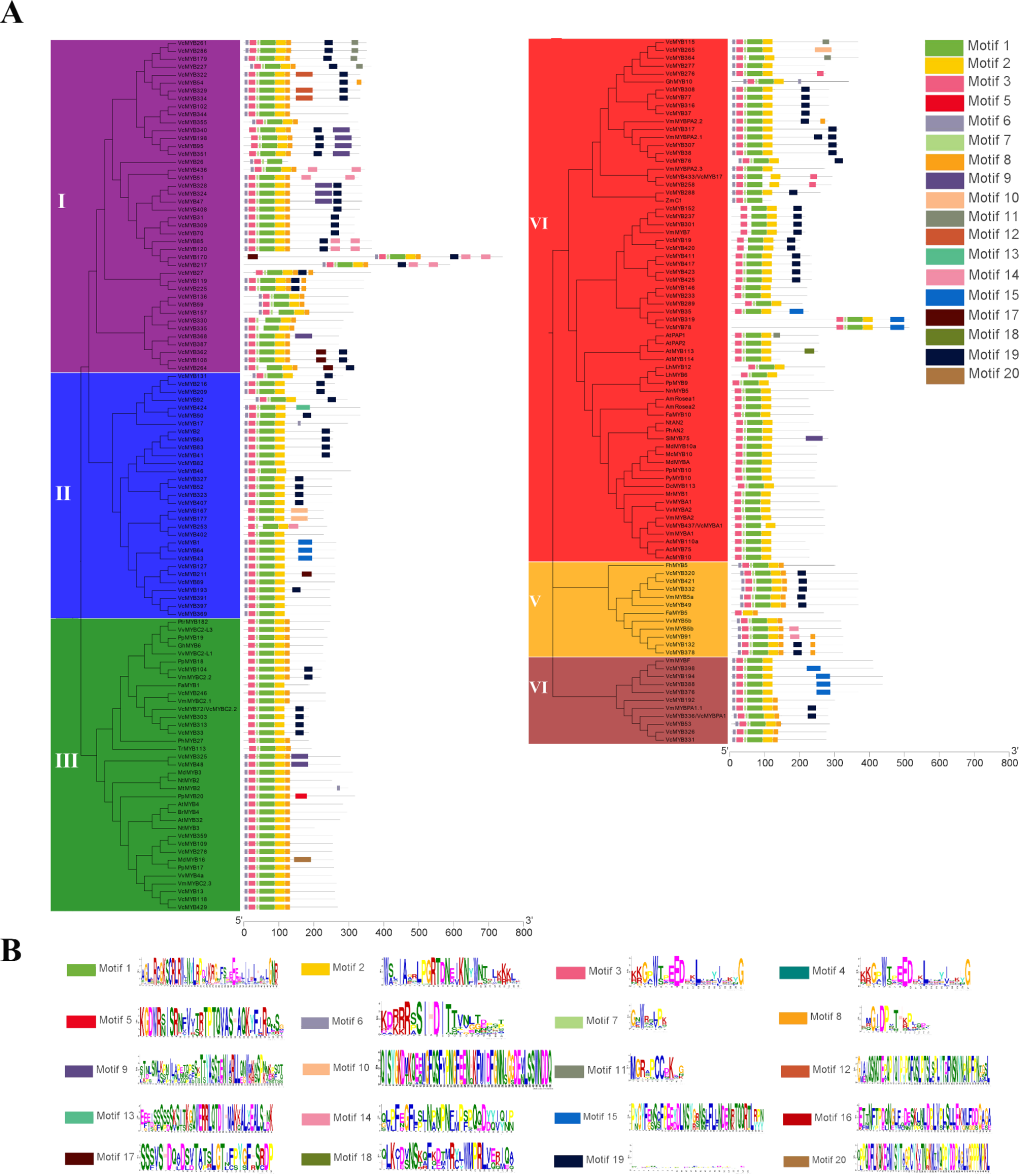
The conserved motif compositions of VcMYB proteins. **(A)** The motif compositions of VcMYB proteins in phylogenetic tree1. **(B)** The sequence logos of 20 motifs. These logos of the R2 and R3 VcMYB repeats were based on multiple full-length alignments of all blueberry R2R3-MYB domains. The bit score represents the information content for each position in the sequence. Asterisks represent the conserved residues that are identical among all R2R3-MYB domains, and triangles denote the typically conserved residues (Trp) in the R2R3-MYB domains. Gly (G), Glu (E), Asp (D), Cys (C), Arg (R), Leu (L), Iie (I), Thr (T), Asn (N) and Lys (K) The height of the letters within each stack indicates the relative frequency

A MEME online program analysis of the conserved motifs of VcMYB proteins was performed to further analyze the characteristics of these R2R3-MYBs. 20 motifs, named motif 1–20, were determined for these MYB proteins (Fig. S2A, B, Fig. 3). As shown in Table S1, the pivotal conserved domains of MYB were identified as motif 1, 2, 3 and 4, and these motifs were taken together to form the SANT domain. Meanwhile, on the basis of the prediction, the conserved motifs, motif 1, 2, 3 and 19, were present in the most conserved genes with 411, 420, 487 and 474, while motif 17 and 20 were present in the least conserved genes with just 9. Motif 4, 9,10,12 and 20 composed of 50 amino acids, but motif 7 had only 8 amino acids (Table S3). Of course, several VcMYB proteins which were clustered into the one polygenetic subgroup were usually detected with the similar motif composition (Fig. 3A). For instance, phylogenetic tree1 subgroup clade I-VI contained motif 1, 2 and 3, demonstrating the functional similarity in the same subgroup [55, 56].

In the VcR2R3-MYBs, the R2 and R3 MYB domains contained typical tryptophan (Trp), a critical amino acid for binding DNA to specific sequences, with many other conserved amino acids, too [55]. R2 and R3 repeats were constructed by motif 3, motif 7, motif 1 and motif 2 (Fig. 3B). Inside of the two MYB domains, we identified 5 conserved Trp residues. In motif 1, the conserved Trp residue was generally replaced with other amino acids (Fig. 3B), meanwhile, the first and second conserved Trp residues were identified in the motif 2 in blueberry. Moreover, R2R3-MYBs also contain other highly conserved amino acids, such as Arg (R), Leu (L) and Lys (K) (Fig. 3B).

### Conserved domain analysis of VcMYBs

**Fig. 4:**
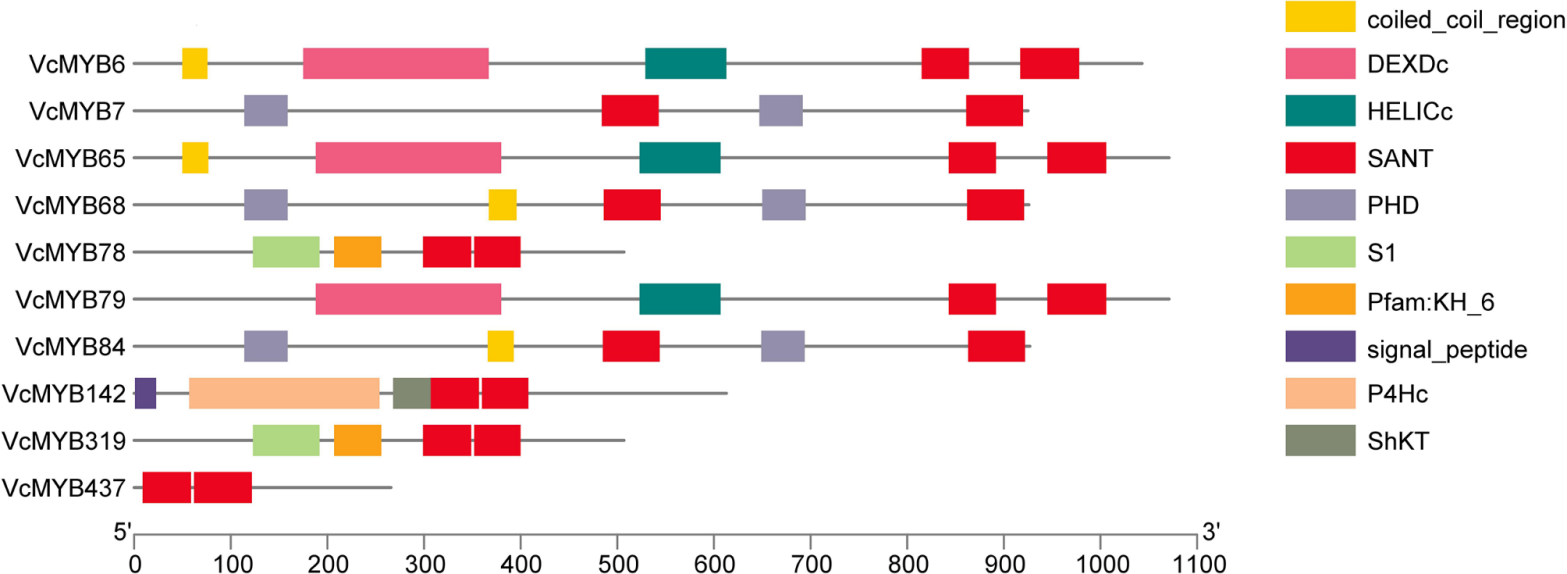
The conserved domains of VcMYB proteins. The symbols are corresponding to the colored box in the right panel

To obtain information about the functional diversity of VcMYB family, SMART online tools were used to analyze their domain components. Consequently, all the 437 family members from VcMYB1 to VcMYB437 contained two SANT domains meaning that they are R2R3-MYBs (Table S1). However, 9 VcMYBs proteins from VcMYB6 to VcMYB319 showed in Fig. 4 contain other domains. For example, VcMYB6, VcMYB65 and VcMYB79 contain DEXDc and HELICc domains, they are all represents the DNA-binding domain of classical SF1 and SF2 helicases, meaning while the HELICc domain is helicases participating in the mechanisms by which these proteins catalyze the remodeling of DNA and RNA in ATP-dependent activities [57, 58], while VcMYB78, VcMYB319, VcMYB84 and VcMYB142 harbor KH_6, PHD, S1, signal peptide, ShKT and P4Hc, and P4Hc is Prolyl 4-hydroxylase alpha subunit homologues, which might catalyze hydroxylation of antibiotic peptides [59].

### *VcMYB* family exhibits diversity expression patterns during fruit development

**Fig. 5:**
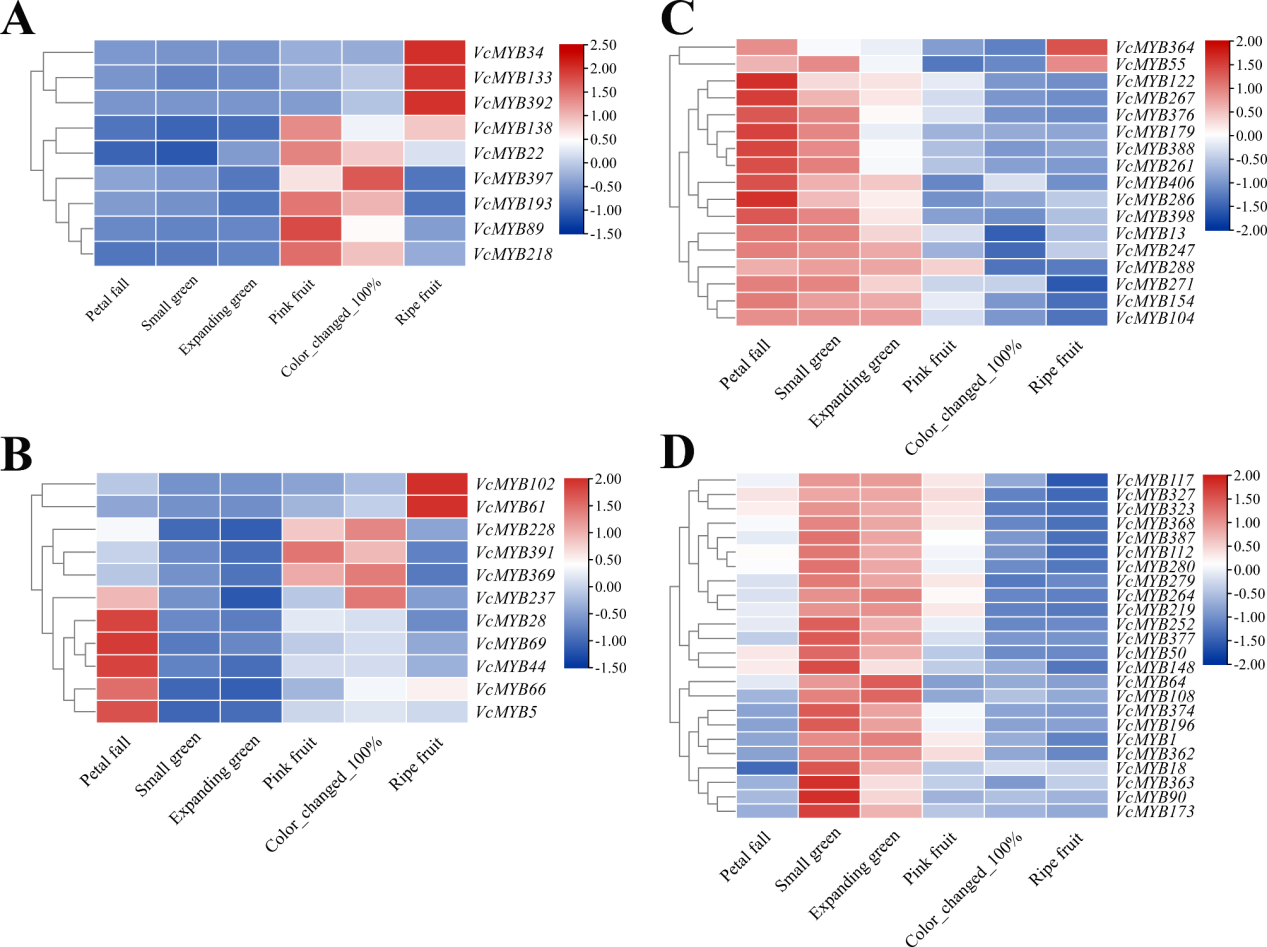
Expression analysis of *VcMYB* genes during fruit development. Transcript profiling of 61 differentially expressed *VcMYB* genes between the stage of Small green or Expanding green and the phase (Pink fruit and Color_change_100%) at the same time. **(A, B)** 20 *VcMYB* genes showed relatively high accumulation in later stages (Pink fruit, Color_changed_100% and Ripe fruit). (A) 9 *VcMYBs* in group I has been a gradual rise from Petal fall to Color_change_100%. (B) 11 *MYBs* in group II have been a relatively fall from Petal fall to Small green, and then gradually rise from Small green to Color_change_100%. **(C, D)** 41 *VcMYB* genes showed relatively high accumulation in early stages (Small green, Expanding green). (C) 17 *VcMYBs* have been a steady fall from the first period (Petal fall stage). (D) 24 *VcMYBs* expressed decreasingly from Expanding green to Color_change_100% whereas increasingly from Petal fall to Expanding green. The transcriptome data from different developmental stages (Petal fall, Small green, Expanding green, Pink fruit, Color_changed_100% and Ripe fruit) were extracted from the previous study reported by Marivi et al. [60]. The color scale beside the heatmap indicates gene expression levels, low transcript abundance indicated by blue color and high transcript abundance indicated by red color

To explore the roles of *R2R3-MYB* family in flavonoid accumulation during fruit development, in this study, we retrieved the publicly available transcript profiling data of gene expression throughout fruit development in blueberry fruit, maturation, and ripening (six stages: Petal fall, Small green, Expanding green, Pink fruit, Color_changed_100% and Ripe fruit) [60]. According to the above screening, the fruit development transcript profiling data of 347 *VcMYBs*, originated from the 437 *R2R3-MYB* genes, we totally identified in the present study, were extracted from the dataset fortunately and shown in Fig. S3A, B, C. Further analysis with more strict screening criteria showed that totally 61 *VcMYB* genes were identified which 2-fold differentially expressed between the developmental stages of early periods (Small green and Expanding green) and maturation phase (Pink fruit and Color_change_100%), the relative expression of these 61 *VcMYB* genes was illustrated in Table S4-1. Four groups of *VcMYBs* were classified based on their transcript accumulation patterns (Fig. 5). The *VcMYBs* in the group I (Fig. 5A) and II (Fig. 5B) showed predominantly high transcript abundance at maturation phase (Pink fruit and Color_change_100%), whereas relatively low and stable accumulation in early periods (Small green and Expanding green). As shown in Fig. 5A, the group I consists of 9 *VcMYBs*, including *VcMYB34*, *VcMYB133*, *VcMYB392*, *VcMYB138*, *VcMYB22*, *VcMYB397*, *VcMYB193*, *VcMYB89* and *VcMYB218*, the transcript abundance of which rose gradually from Petal fall to Color_change_100%. While 11 *VcMYBs* in group II had a relatively fall from Petal fall to Small green as early fruit developments, and then gradually rose from Small green to Color_change_100%, such as *VcMYB237*, *VcMYB391*, and *VcMYB369* (Fig. 5B). Notably, *VcMYB391*, *VcMYB369*, and *VcMYB218* in group I and II showed a remarkable increase from Expanding green to Pink fruit which is roughly 67, 57, 33-fold, respectively (Table S4-2, S4-3). Although, the changes of several *VcMYBs* were not as obvious as much in Figures, such as *VcMYB133, 138* and *61* (~ 2 to 4-fold, Table S4-3, S4-4), their expression abundance was actually significantly difference in their expression data. It was noticed that *VcMYB152*, *VcMYB237* and *VcMYB301* showed high homology to flavanol biosynthesis activators (Fig. 2A), *VmMYB7*, for instance, which are proved to activate transcription of anthocyanin biosynthetic genes [26, 47], implying their functional similarity. Moreover, as shown in Fig. S3A, four genes, *VcMYB335*, *VcMYB31*, *VcMYB70* and *VcMYB152*, grouped in phylogenetic tree1, were transcriptionally elevated during fruit development from Expanding green stage to Color_change_100% stage, but their expression in the stage of Small green were quite high, too (Fig. S3A).

Interestingly, several *VcMYBs* displayed different, even opposite, accumulation patterns. The *VcMYBs* in the group III (Fig. 5C) and IV (Fig. 5D) had relatively low and stable expression in mature periods, whereas relatively high accumulation in early development phases. Totally, 17 *VcMYBs* in group III (Fig. 5C), for instant, *VcMYB247*, *VcMYB271* and *VcMYB104*, had been a steady fall from the first stage (Petal fall stage). Similarly, 24 *VcMYBs* in group IV (Fig. 5D) expressed decreasingly from Expanding green to Color_change_100%, whilst increasingly from Petal fall to Expanding green, such as *VcMYB219*, *VcMYB280* and *VcMYB1*. While together with these *VcMYBs* in group III and IV, *VcMYB377*, *VcMYB64* and *VcMYB406* were transcriptionally decreased when fruit ripens, with the maximum expression ratio of Expanding green/Pink fruit, 19, 21, 20-fold, respectively (Table S4-2, S4-3, S4-4). Moreover, besides these 41 *VcMYBs* which showed higher expression in fruit early developmental period, 20 *VcMYBs* were derived in phylogenetic tree1 with activating transcription factors and transcription-repressors, such as *VcMYB13*, *VcMYB327*, *VcMYB368* and *VcMYB398*.

**Fig. 6:**
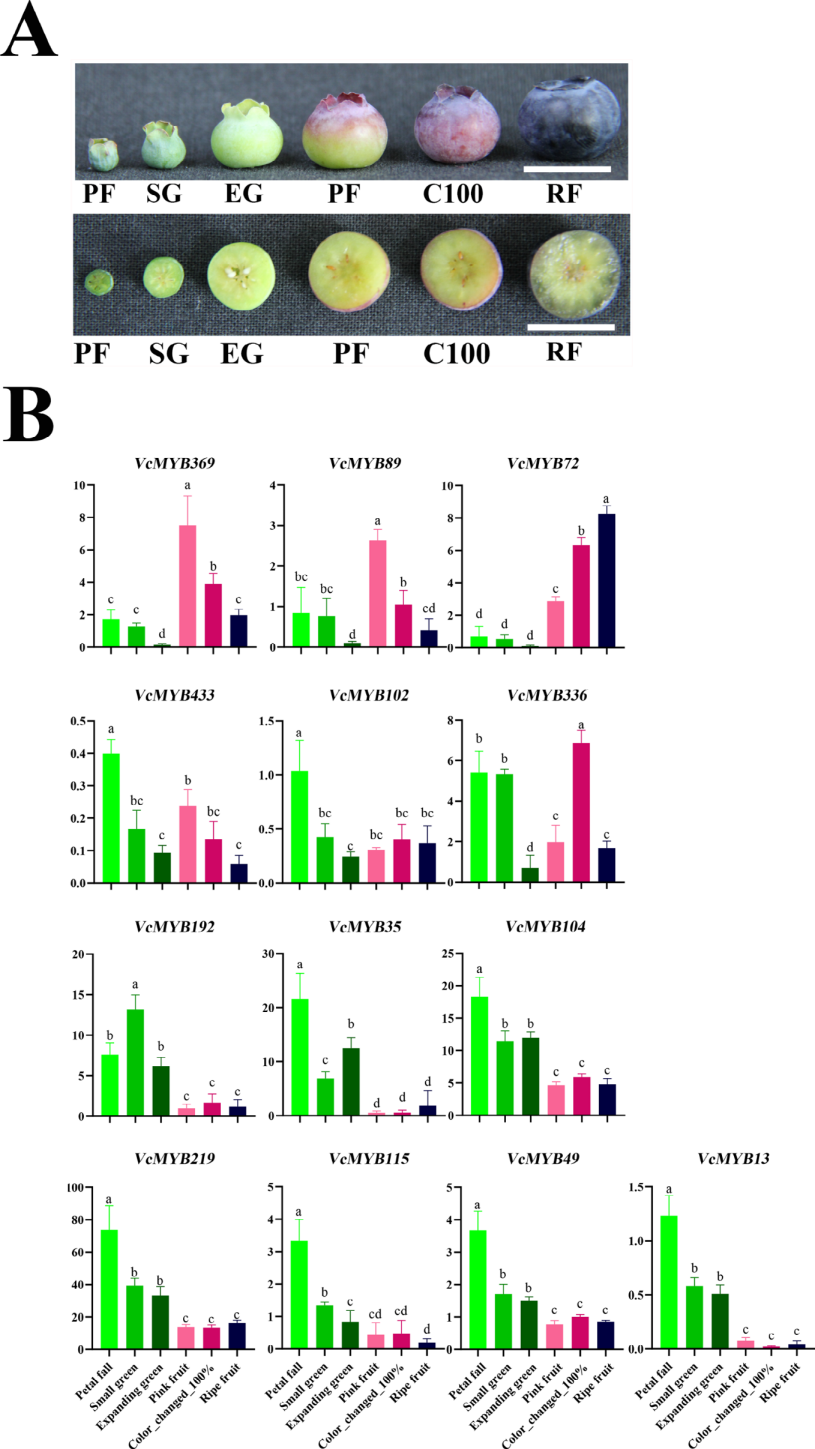
Analysis of *VcMYB*s underlying fruit development. **(A)** Photograph of fruits and cross-section of fruits at six developmental stages [Petal fall (PF), Small green (SG), Expanding green (EG), Pink fruit (PF) Color_changed_100% (C100) and Ripe fruit (RF)], the scale bar indicates 1 cm. **(B)** Expression patterns of *VcMYB*s during fruit development. Total RNAs were extracted from fruit of the cultivar ‘Northland’ (*V. corymbosum*) during six different stages in fruit. Data were normalized against *VcGAPDH*. Error bars indicate SE of three biological and technical replicates, and different letters indicate significant difference (*P* < 0.05)

Based on the amount of expression and position on the phylogenetic tree, 13 *VcMYB*s were selected to investigate their expression profiles underlying blueberry fruit development [six phases from the earliest stage (Petal fall) to the final stage (Ripe fruit), Fig. 6A] by qRT-PCR approach. As shown in Fig. 6B, six genes were transcriptionally increased at maturation phase (Pink fruit and Color_change_100%) compared with the Expanding green period. Interestingly, *VcMYB433*, *VcMYB102* and *VcMYB336* showed high expression both in maturation phase and early development (Petal fall and Small green). In contrast, a relatively fall expression trend from early fruit development was observed on seven *VcMYB*s with the maximum expression at either Petal fall or Small green stage, including *VcMYB192*, *VcMYB35*, *VcMYB104, VcMYB219*, *VcMYB115, VcMYB49* and *VcMYB13* (Fig. 6B).

### *VcMYB* family shows different transcript profiles between skin and pulp in fruit

**Fig. 7:**
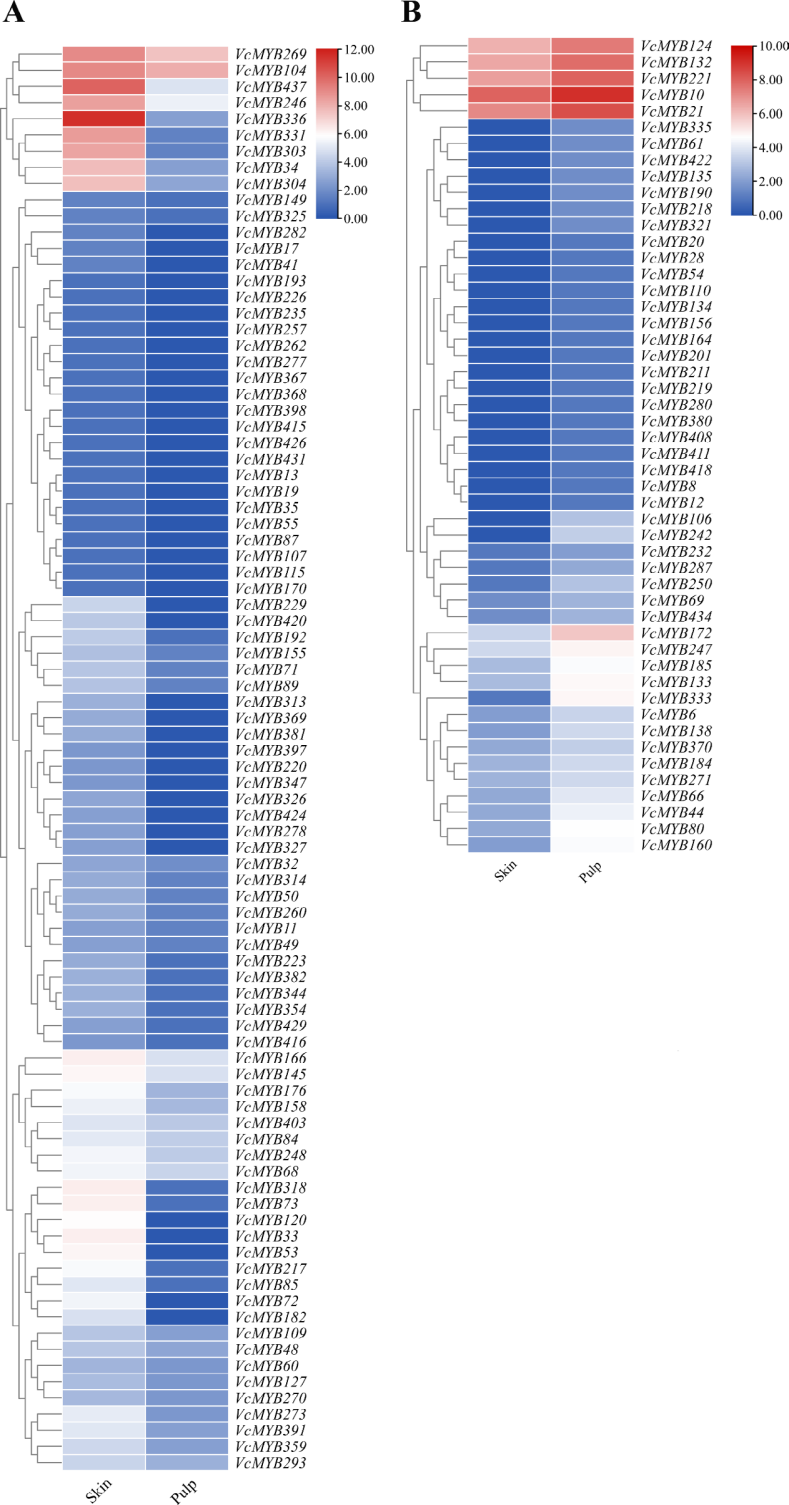
Expression analysis of *VcMYB* genes in skin and pulp. Transcript profiling of 138 differential expressed *VcMYB* genes in skin and pulp. **(A)** a total of 88 *VcMYB* genes showed relatively high accumulation in skin. **(B)** 50 *VcMYB* genes showed relatively high accumulation in pulp. The transcriptome data from skin and pulp were obtained from the previous study reported by Li et al. [81]. The color scale beside the heat map indicates gene expression levels, low transcript abundance indicated by blue color and high transcript abundance indicated by red color

**Fig. 8:**
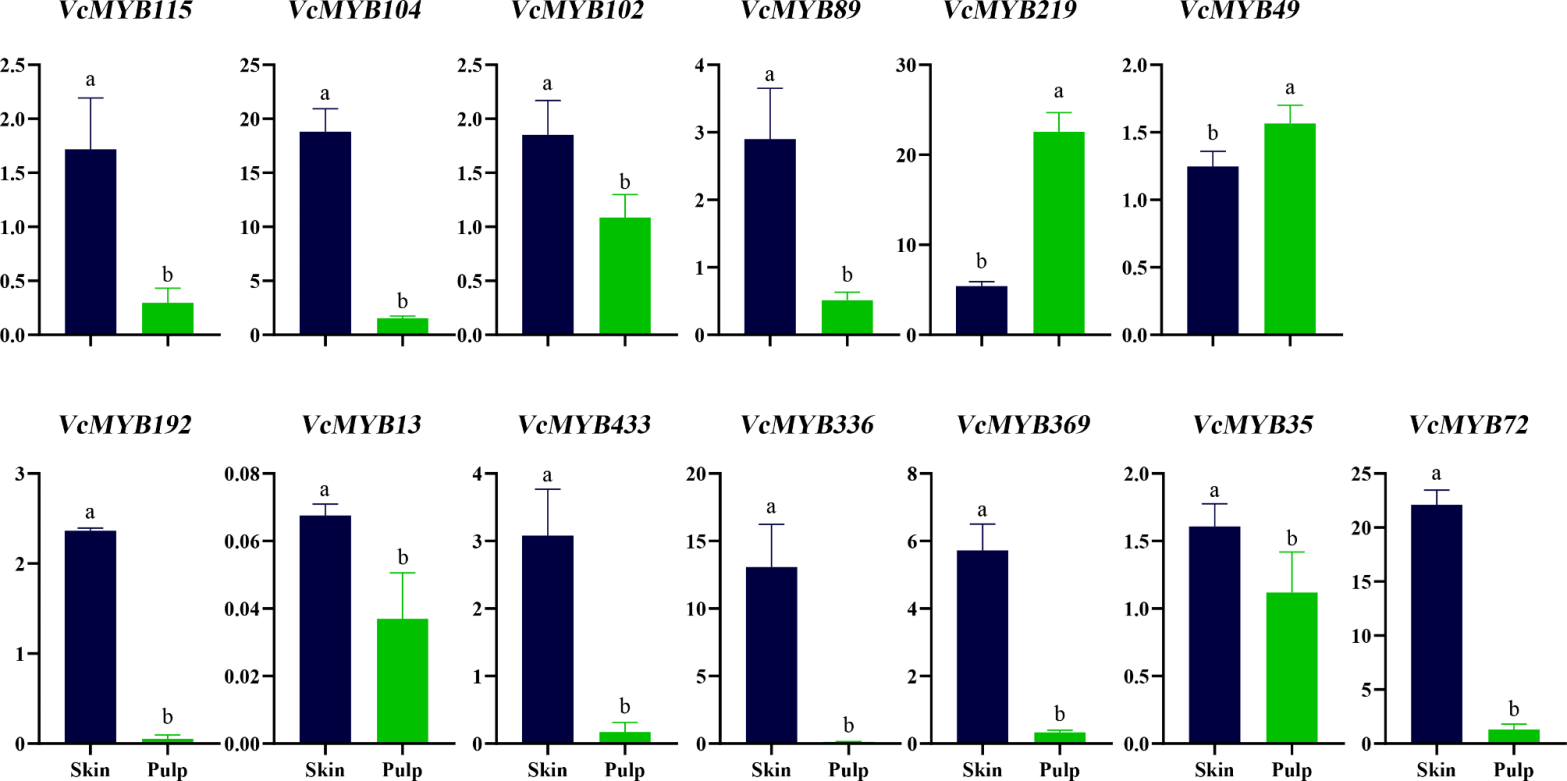
Expression patterns of *VcMYB*s in different tissues of ripen fruit. Total RNAs were extracted from fruit of the cultivar ‘Northland’ (*V. corymbosum*) during Ripe fruit stage. Data were normalized against *VcGAPDH*. Error bars indicate SE of three biological and technical replicates, and different letters indicate significant difference (*P* < 0.05)

Anthocyanins are produced in a variety of tissues in blueberries, and anthocyanins are mainly found in the skin of the fruit and the flesh is primarily acyanic. Based on the accumulation patterns of *VcMYB* genes between skin and pulp, it was observed that 88 *VcMYB* genes displayed higher accumulation in pericarp (Fig. 7A), whereas altered transcript profiling was observed for 50 *VcMYB* genes in Fig. 7B. A total of 9 *VcMYB*s were highly expressed in blueberry skin, including *VcMYB269*, *VcMYB104*, *VcMYB437*, *VcMYB246*, *VcMYB336*, *VcMYB331*, *VcMYB303*, *VcMYB34* and *VcMYB34*, with the maximum expression from *VcMYB437* and *VcMYB336*, while relatively low accumulation was observed in the pulp (Fig. 7A, Table S4-5). This further evidence suggests that the 9 *VcMYBs* are involved in anthocyanin synthesis. Additionally, *VcMYB33*, *VcMYB53*, *VcMYB336* (*VcMYBPA1*) and *VcMYB120* transcriptionally increased in skin more than 590-fold compared to expression in the pulp. By contrast, as shown in Fig. 7B, *VcMYB242*, *VcMYB106* and *VcMYB333*, exhibited a dramatically drop accumulation in pulp, which decreased approximately 90-, 70-, 27-fold respectively (Fig. 7B, Table S4-5). Subsequently, by qRT-PCR approach, 13 *VcMYB*s were chosen to verify the transcriptional expression pattern between skin and pulp in ripen fruit. As shown in Figs. 8 and 11 *VcMYB*s all had a relatively high accumulation in the blueberry skin. Especially, the expression level of *VcMYB336* in skin were 105.42-fold of the one in the pulp, presenting particularly significant differences, which is consist with the transcript profiling in Fig. 7A. In contrast, *VcMYB219* and *VcMYB49* were mainly expressed in fruit pulp, nevertheless, the fold change between the two tissues no more than 4.56-fold (*VcMYB219*, Fig. 8).

To comprehensively understand the role of *VcMYB*s in anthocyanin biosynthesis in blueberry, the analysis of the association upon accumulation patterns between the fruit development and different tissues (skin and flesh) was conducted. Consequently, by the criteria of greater than 2-fold differences, six *VcMYB*s were explored, comprising of *VcMYB369*, *VcMYB391*, *VcMYB193*, *VcMYB397*, *VcMYB89* and *VcMYB34*, which represented strongly upregulated underlying the mature phase as well as in the skin, simultaneously (Figs. 5A and 7A), and clustered in phylogenetic tree 1, exception for VcMYB34, which fell into subgroup VI of phylogenetic tree 2 with a 41-fold differential between skin and flesh (Fig. S1A, Table S4-5). In addition, several *VcMYB*s in greater abundance in skin and developmental phase were also in the spotlight in this study, including *VcMYB13*, *VcMYB327*, *VcMYB368*, *VcMYB398*, *VcMYB50*, *VcMYB104* and *VcMYB55*. Of these, *VcMYB13* and *VcMYB104*, both in phylogenetic tree 1, were divided into the same clade with the *MYB*s from other dicot species, like VmMYBC2.2/2.3, VvMYB4a/C2-L1/C2-L3 and PpMYB18 (Fig. 2), which act as the repressors associated with anthocyanin regulation [40]. Noticeably, 4 (*VcMYB271*, *VcMYB247*, *VcMYB219* and *VcMYB280)* out of 41 *MYB*s in groups III and IV with higher expression in fruit early developmental period also showed more accumulation in pulp than skin.

## Discussion

In many fleshy fruit species, it is start to synthesize pigment, such as flavonoids and anthocyanins especially, during ripening [61]. It is also known that several aspects, including light, temperature and nutrients, have an effect on flavonoid accumulation [62]. Additionally, the flavonoid biosynthesis was regulated by the interaction of transcription factors, including basic helix-loop-helix and WD40-repeat proteins [7, 63–65]. However, blueberries are still lack of adequate regulators for the synthesis of important secondary products such as anthocyanins.

A crucial role for R2R3 MYB factors in plant is an important key in development as well as determination of cell fate and identity [66]. The *MYB* gene family has been extensively described in *Arabidopsis thaliana*, Nevertheless, the knowledge about the MYB protein family in blueberry is very limited. According to the present study, 437 *VcMYB*s were identified, far more than those found in *Arabidopsis thaliana* with 125 *AtMYB*s [18]. The blueberry genome size (haploid) is roughly 600 Mb [67], and there are 56,087 annotated genes, which is approximately 2.05 times as many as in the genome annotation of *Arabidopsis thaliana* [68]. Thus, compared to *Arabidopsis thaliana*, blueberry has a much larger *MYB* gene family. Phylogenetic analysis of MYB proteins provided insights into not only the genetic and species-to-species evolutionary relationships, but also putative functional assignments. As shown in Fig. 2 and 62 VcMYBs can be clustered together with the MYBs of other plant species documented as activators of anthocyanin biosynthesis, implying that they are possible to have experienced similar evolutionary diversification and share similar functions. Notably, as shown in Fig. 1 and Table S2-2, there are a large number of digenic, trigenic and tetragenic *VcMYB* pairs showing high identity (over 95% at nucleotide level) with each other, suggesting that they undergone restrictively functional diversification. In general, gene families emerge from gene duplication during evolution, resulting in neofunctionalizations and backup genes [69]. Blueberry species went through at least three rounds of whole genome duplication during evolution [70], and multiple copies of genes were supposed to be generated in this way. Thus, in blueberry, it was deduced that with the early gene duplication events of chromosomal segment as well as whole genome, the expansion of *MYB* genes had occurred.

Increasing number of MYBs are proved to be involved in anthocyanin biosynthesis in various colored tissues of plant, since the first plant MYB protein controlling anthocyanin synthesis was investigated in maize (*Zea mays*) [70, 71]. For instance, overexpression of apple *MdMYB10a* in *Nicotiana tabacum*, which was clustered in the subgroup VI of phylogenetic tree (Fig. 2), promoted the anthocyanin accumulation by activating the expressions of anthocyanin-biosynthetic genes and corresponding regulators [72]. Here, 61 out of 437 *VcMYB*s were differentially expressed as fruit grows and/or ripens. Of these, 20 *VcMYB*s showed higher expression in mature phase, whereas 41 displayed the opposite expression pattern. Moreover, in blueberry, a great abundance of anthocyanins is extremely restricted to the fruit skin, whereas only trace amounts are detected in fruit pulp [42, 47]. Here, totally 88 *VcMYB*s showed transcript accumulation in fruit skin more than in pulp (Fig. 7 ), suggesting that they might play important roles in fruit skin such as formation and deposition of pigments. Intriguingly, it was observed that *6 VcMYBs*, including *VcMYB397*, *VcMYB193*, *VcMYB391*, *VcMYB369*, *VcMYB89* and *VcMYB34*, were highly expressed in both fruit ripening period and skin with the maximum differences of 67-fold (a 6.07 log2fold differential, Figs. 5A and B and 7A) as compared with the ones in fruit pulp and the early stages of fruit development, in accordance with the expression level of *MYBA* (gene model 38,459) between the pigmented skin and acyanic flesh of the berry [1]. In addition, exception of *VcMYB89 and VcMYB34*, the four *VcMYB*s (*VcMYB397*, *VcMYB193*, *VcMYB391* and *VcMYB369*) were all clustered together in phylogenetic tree 1, which showed with more than 97% identity to each other, indicating that they might be ortholog genes (Fig. 2). These results suggested that the four *MYB*s probably act as key components of anthocyanin production pathway during blueberry fruit development, which needs to be further studied in the near future. Recently, it is reported in bilberry, when *VmMYBPA1.1* is silenced, fruit loses anthocyanins due to the activity repression of *CHS*, *DFR*, and *ANS* promoters [47, 73]. Here, the tetragenic *VcMYB* pairs, *VcMYB53*, *VcMYB326*, *VcMYB331* and *VcMYB33*6, exhibited very close relationship with *VmMYBPA1.1* in the clade VI of the phylogenetic tree (Fig. 2). Noticeably, the four *VcMYB*s showed increased expression in fruit skin with high anthocyanin accumulation but quite low expression in pulp (Fig. 7), which is consistent with the ones of *VmMYBPA1.1* in bilberry [47, 54]. More importantly, *VcMYBPA1.1* gene expression profiles show peaks during early and late stages of development, when proanthocyanidins (PA) biosynthesis occurs and anthocyanins accumulate, respectively [42]. Furthermore, VcMYBA1 and VcMYBPA1 positively co-regulate the accumulations of PAs and anthocyanins by activating *VcbHLH2* and *VcMYBC2.1* [43, 47, 54]. The protein sequences of VcMYBA1 and VcMYBPA1 were highly homology to VcMYB437 and VcMYB336, respectively, in this study (Fig. 2), implying they might functionally similar. In blueberry flesh, *VcMYB336* had very low or barely detectable expression, while it was considerably highly expressed in skin (Figs. 7 and 8), although no significant difference (~ 1.88-fold, Table S4-2) was identified between the accumulation of Pink fruit stage and Expanding green stage of *VcMYB336*, having similar developmental expression profiles of *VcMYBPA1.1 * [54]. *VcMYB437* (probably homologous to *VcMYBA1*) was in greater abundance in skin. As proven that VcMYBA conducts as an anthocyanin modulator activating promoter of the anthocyanin biosynthetic gene *DFR* and the probable central activator of fruit skin pigmentation [1]. Thus, it is worth to explore if *VcMYB336* and *VcMYB437* playing the same role in anthocyanin production as well as the downstream targets involved in the pathway. All the clues imply that these *VcMYB*s might work as important roles in anthocyanins accumulation as well as activate the downstream genes similar to other plant species, providing further data getting behind the regulation of TF genes involved in anthocyanin regulation.

In contrast to these highly conserved core R2R3-MYB activators, a fairly large number of suppressor factors involved in anthocyanin accumulation have also been well documented, mostly in the last decade. Noticeably, several *VcMYB*s, such as *VcMYB104* and *VcMYB13* (Fig. 5C), showed down-regulated expression pattern as fruit develops, the same as the R2R3-MYBs with a previously characterized role in negatively regulating anthocyanin production [1], suggesting that they might contribute to suppress the biosynthesis of anthocyanin as well as those MYB suppressors. It was deserved to be mentioned that *VcMYB13*, *VcMYB104* and tetragenic VcMYB pairs (*VcMYB72/VcMYBC2.2, VcMYB33, VcMYB303 and VcMYB313*) were clade into subgroup III with *VmMYBC2.3, VvMYB4a*, *VmMYBC2.2 and VmMYBC2.1*, respectively, (Fig. 2) which inhibit anthocyanin accumulation [21, 40, 43, 74, 75], and showed higher expression in fruit early developmental period [43, 47] or more abundant in the skin (Fig. 7). It was consistent with the expression tendency of *VcMYBC2.1* (SG4 R2R3-MYBs with TLLLFR motifs contributing to repressive activity) and *MYBR3.1* (R3 MYB, small proteins lacking activation and repression motifs), both of which are well characterized as MYB repressors with distinct functions underlying anthocyanin production [37, 43, 54, 76]. Combined with previous studies, we know that MYBA1 can activate the promoters of *MYBC2.1* and *bHLH2* by hierarchically regulation, when co-infiltrated of *MYBC2.1* and *MYBA1*, anthocyanins were substantially reduced, and so did the activation of *DFR* and *UFGT*, which are widely recognized as key anthocyanin biosynthesis genes [43]. Thus, in the further study, it is worth to explore the regulatory network among *VcMYB336* (homologous to *VcMYBPA1*), *VcMYB437* (homologous to *VcMYBA1*), *VcMYB72* (homologous to *VcMYBC2.2*) and other core gene correlated strongly with anthocyanin biosynthesis.

Combined with these results, the regulatory network of PA and anthocyanin biosynthesis remind confusions, demanding coordination of both activator and repressor transcription factors. At present, the reported MYB TFs closely related to anthocyanin biosynthesis in blueberry fruit were mainly concentrated in the MYBs discussed previously, and the function for a large number of candidates MYBs in our study are necessary to be further explored. This research will provide new promising candidates for future study on improving the coloration level of fruit and giving new insight into the regulatory mechanisms for anthocyanin biosynthesis in blueberry.

## Conclusions

In conclusion, 437 unique VcMYB sequences were identified in blueberry, which were grouped into three phylogenetic trees. The combination analysis of motif architectures, phylogenetic relationships, and sequence identity showed that the functional roles and features of VcMYB family are conserved and divergent across plant species. Furthermore, their expression patterns and transcript profiles suggested that *VcMYB* family might contribute to the regulation of fruit development and the specificity of fruit skin and pulp. Their authentic roles and regulatory mechanisms in blueberry still need to be documented in future studies.

## Methods

### Identification of *VcMYB* family genes

From NCBI (www.ncbi.nlm.nih.gov) database, we obtained the publicly available MYB protein sequences in bilberry, grape, apple, pear, petunia, peach, kiwi, snapdragon, carrot, fragrant sherry, strawberry, cotton, corn, bayberry, lotus, narcissus, tomato, Arabidopsis, poplar and lily. All sequences of the complete transcripts were extracted from the database of *Vaccinium corymbosum* GDV RefTrans v1 (www.vaccinium.org). In the Pfam database (http://pfam.xfam.org/), the hidden Markov model (HMM) file (PF00249) of MYB domain was downloaded, HMMER for scanning and identification, and its cut-off e-value is 0.01 [77]. Subsequently, to confirm two intact MYB domains were in the MYB sequences, all candidate VcMYBs were verified using SMART (http://smart.embl.de/) [78] and Pfam Program Following this, all potential hits were collected for *R2R3-MYB* genes that contain conserved regions. Additionally, all potentially redundant VcMYBs sequences were discarded after analyzing the remaining sequences with ClustalX software. Sequences that are non-redundant have been renamed according to their gene number in the *Vaccinium corymbosum* GDV RefTrans v1 database. For each VcMYB, the theoretical isoelectric point (PI), molecular protein weight (kDa) and an average of hydropathicity (GRAVY) index was obtained by the ProtParam tool (https://web.expasy.org/protparam/).

### Chromosomal locations of *VcMYBs* and phylogenetic analysis of *MYB* genes

In order to determine their location on the genome of *Vaccinium corymbosum* (Draper), the *VcMYB* genes were mapped to the genome of this plant. Subsequently, we imported the information about their identities (95%), scaffold positions, and scaffold positions into the software TBtools to generate a circle plot [79]. The Amino acid sequences of MYBs (13 from bilberry, 8 from Arabidopsis, 7 from apple, 6 from grape and peach, 3 from kiwi, snapdragon, strawberry and narcissus, 2 from cotton, petunia and lily, 1 from pear, carrot, fragrant sherry, bayberry, lotus, tomato, corn and poplar) were useed to build phylogenetic trees with VcMYBs. Briefly, multiple sequences alignment of all the MYBs were performed by Clustal Omega (https://www.ebi.ac.uk/Tools/msa/clustalo/), and phylogenetic trees were constructed by the MEGAX software (https://www.megasoftware.net) with maximum likelihood statistical method [80]. Using 1000 replicates, bootstrap values were calculated in these polygenetic trees.

### Gene conserved motifs and domains of VcMYBs

The conserved motifs of VcMYB proteins were analyzed using the online program Multiple Expectation Maximization for Motif Elucidation (http://meme-suite.org/tools/meme), with the following parameters: the number of different motifs set as 20, and the minimum motif and maximum motif windows set to 6 and 50, respectively. The conserved domains were analyzed using the online program SMART (http://smart.embl.de/), and were visualized by Tbtools.

### Expression of *VcMYBs* during fruit development and in fruit tissues

A detailed understanding of *VcMYB* gene expression underlying fruit development and across tissues (pulp and skin) is necessary. The raw RNA sequencing data was download from NCBI Sequencing Read Archive (SRA, https://www.ncbi.nlm.nih.gov/sra), SRA046311 for different tissues (pulp and skin) [81]. We first qualified raw data in fastq format using the FastQC program, and then processed these reads in Hisat2 version 2.2.124 for read alignment to the genome of *Vaccinium corymbosum* (Draper). The expression value of genes and transcripts were obtained via the conversion to fragments per kilobase of transcript per million fragments mapped (FPKM). In-house R scripts were employed to analyze gene expression as well as heatmap generation. All the heatmaps were generated by the software TBtools, but the transcript profiling data from six stages were specially treated using “Row Scale” in TBtools to better exhibit the difference of expression level between various development stages in Fig. 5 [79]. The *VcMYB* genes with 2-fold differentially expressed were identified as differentially expressed genes. Similarly, transcript profiling data from six stages (Petal fall, Small green, Expanding green, Pink fruit, Color_changed_100% and Ripe fruit) for *VcMYB*s were extracted according to the previous study [60].

Total RNAs were extracted from fruit at six developmental stages (Petal fall, Small green, Expanding green, Pink fruit, Color_changed_100% and Ripe fruit) and separated skin and pulp of ripe fruit of *V. corymbosum* (cultivar ‘Northland’, cultivated in a field at the Horticulture Teaching and Research Center, Jilin University) via RNAprep Pure Plant Plus Kit (TIANGEN, China). cDNAs were generated by StarScript II First-strand cDNA Synthesis Mix With gDNA Remover Kit (GenStar, China). qRT-PCR was performed using the Bio-Rad CFX Connect Real-Time PCR Detection System with the reagent of 2×RealStar Green Fast Mixture (GenStar, China). *VcGAPDH* was set as an internal reference [82]. The data analysis using the Bio-Rad CFX Manager, and three biological replicates with three technical replicates were conducted for each sample. Statistical significance of the data was analyzed using ANOVA with LSD test, Error bars indicate SE and *p*-value < 0.05 was considered to be statistically significant. Primer information is listed in Table S5.

### Electronic supplementary material

Below is the link to the electronic supplementary material.

Supplementary Material 1

Supplementary Material 2

Supplementary Material 3

Supplementary Material 4

Supplementary Material 5

Supplementary Material 6

Supplementary Material 7

Supplementary Material 8

Supplementary Material 9

Supplementary Material 10

## Data Availability

The publicly available RNA sequencing raw data regarding the blueberry fruit skin and pulp were retrieved at SRA of NCBI with accession NO. of SRA046311. The raw data were subsequently qualified in fastq format using the FastQC program, and processed these reads in Hisat2 version 2.2.124 for read alignment to the genome of *Vaccinium corymbosum* (Draper). The expression value of genes and transcripts were obtained via the conversion to fragments per kilobase of transcript per million fragments mapped (FPKM). In-house R scripts were employed to analyze gene expression. All data generated or analyzed during this study are included in this published article [and its supplementary information files].
