# Supplementary figures and images for: Identification of R2R3-MYB family in blueberry and its potential involvement of anthocyanin biosynthesis in fruits
Source: BMC Genomics. 2023 Aug 30;24:505. doi: 10.1186/s12864-023-09605-w (PMC10466896; doi:10.1186/s12864-023-09605-w)

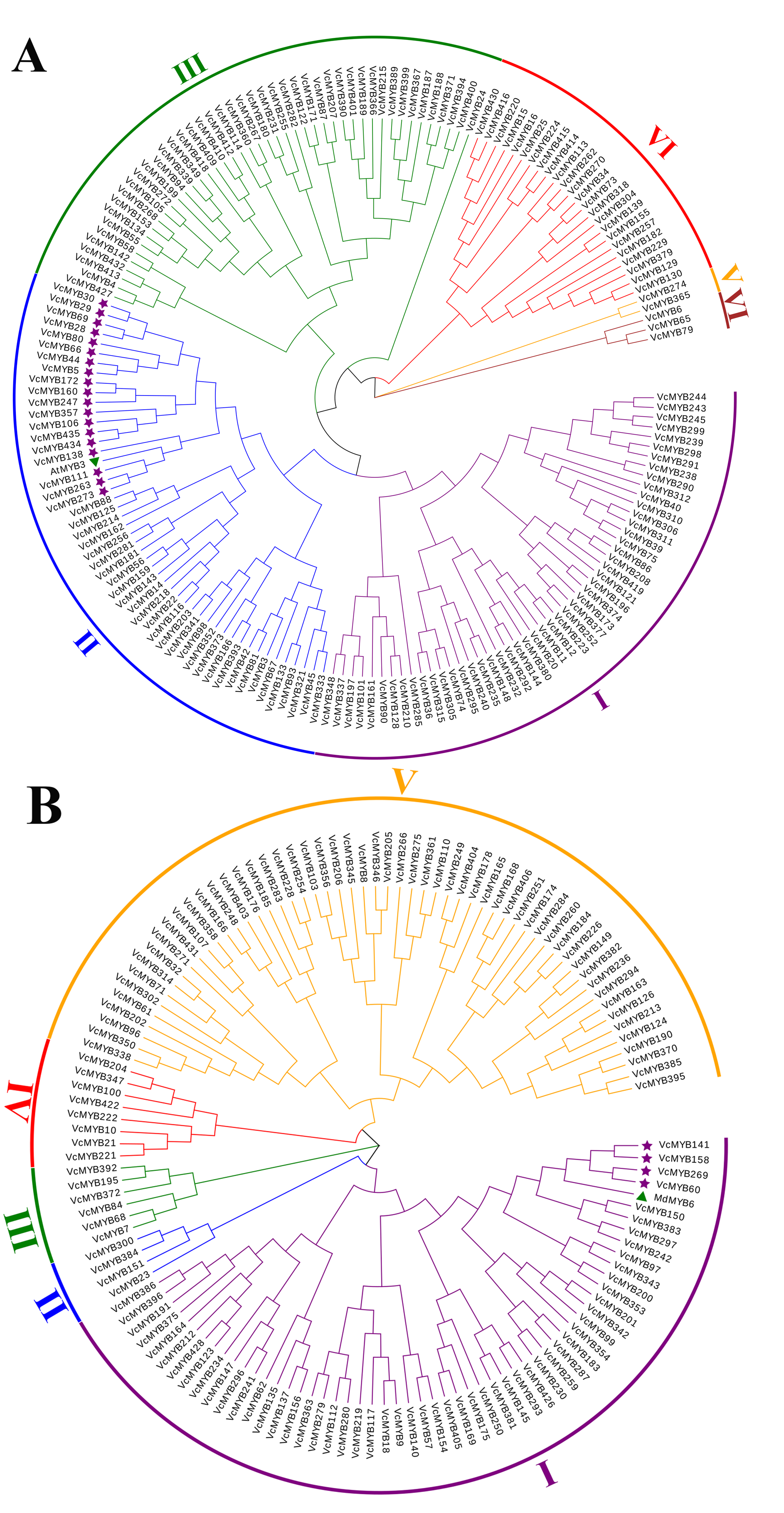

Supplement: Supplementary file 1 — Supplementary Material 1 [file 12864_2023_9605_MOESM1_ESM.tif]

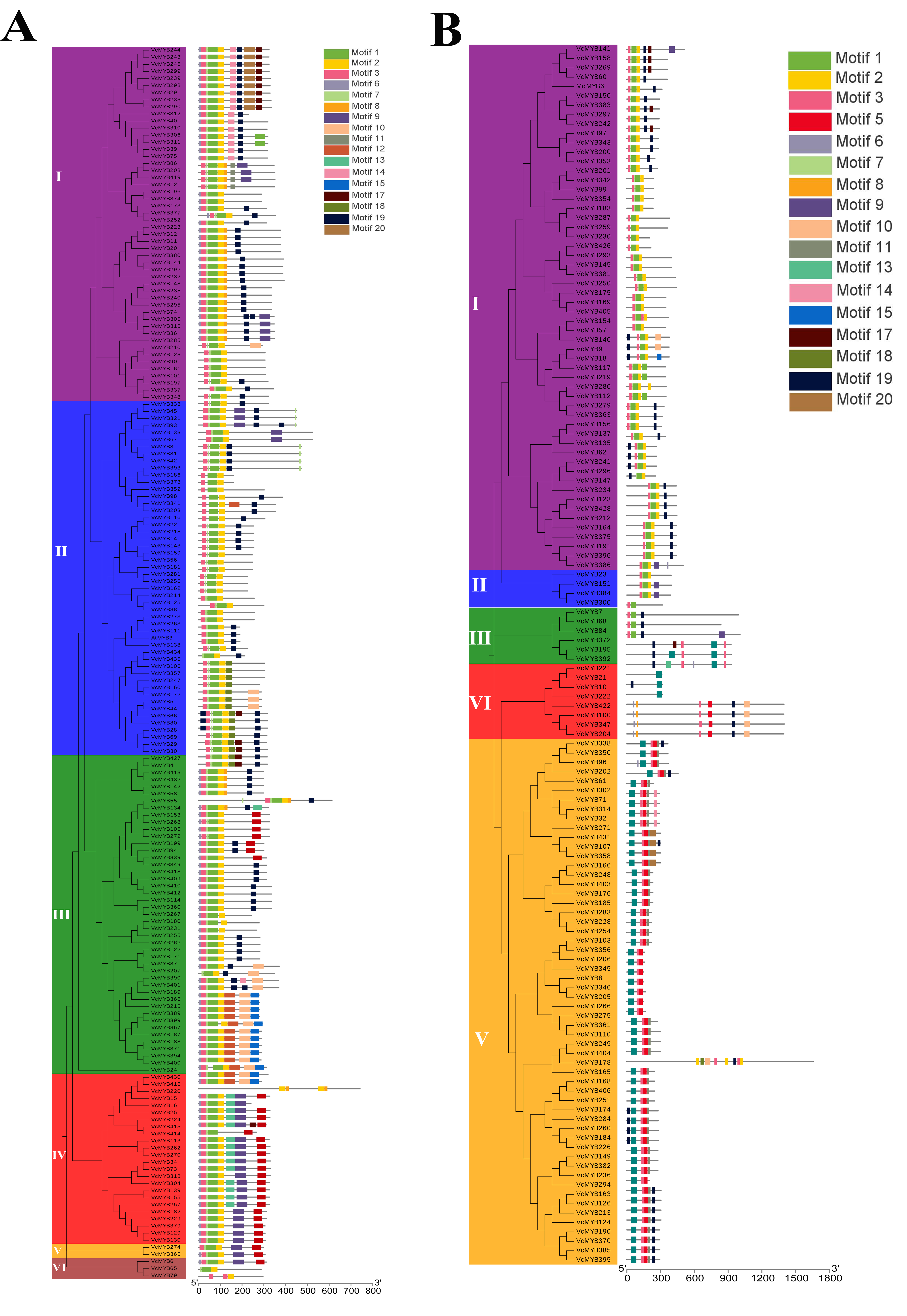

Supplement: Supplementary file 2 — Supplementary Material 2 [file 12864_2023_9605_MOESM2_ESM.tif]

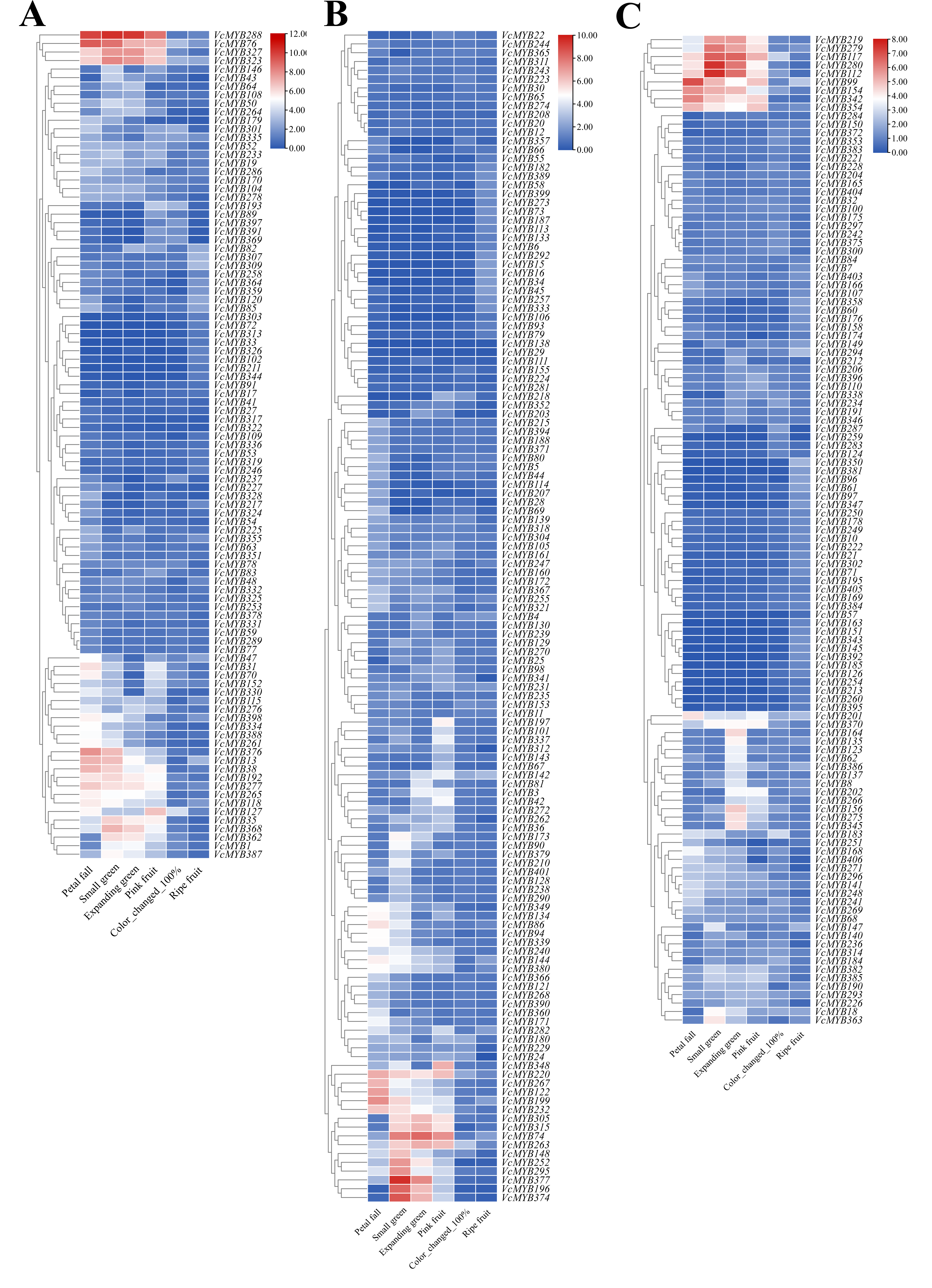

Supplement: Supplementary file 3 — Supplementary Material 3 [file 12864_2023_9605_MOESM3_ESM.tif]
