# Supplementary material for: Identification of R2R3-MYB family in blueberry and its potential involvement of anthocyanin biosynthesis in fruits
Source: BMC Genomics. 2023 Aug 30;24:505. doi: 10.1186/s12864-023-09605-w (PMC10466896; doi:10.1186/s12864-023-09605-w)
Supplement: Supplementary file 9 — Supplementary Material 9 [file 12864_2023_9605_MOESM9_ESM.docx]

**supplementary Figure legends**

**Fig. S1** Phylogenetic relationship of *VcMYB* genes with the R2R3-MYBs in other plant species. The sequences of publicly known MYB genes in apple and arabidopsis were downloaded from NCBI database. 174 MYBs and 130 MYBs were showed in two phylogenetic trees. (A) 174 MYBs were showed in phylogenetic tree 2, and this phylogenetic tree was classified into 6 different groups and clustered together with one MYB from apple. (B) 130 MYBs were showed in phylogenetic tree 3, and this phylogenetic tree was classified into 5 different groups and clustered together with one MYB from Arabidopsis. All the other species R2R3-MYB that can act as repressors of anthocyanin biosynthesis are represented with the same symbol: green triangle, and the VcMYBs which are either divided into one branch with MYBs of other plant species are represented with purple star.

**Fig. S2** (A)The motif compositions of VcMYB proteins in phylogenetic tree 2. (B)The motif compositions of VcMYB proteins in phylogenetic tree 3.

**Fig. S3** Expression analysis of *VcMYB* genes during fruit development. All the expression of 347 *VcMYB*s were divided into 3 phylogenetic trees: tree 1 (A) tree 2 (B) and tree 3 (C), as before. A, B and C Transcript profiling of *VcMYB*s during fruit development. The transcriptome data from different developmental stages (Ripe fruit, Petal fall, Small green, Expanding green, Pink fruit, Color_changed_100%, Unripe fruit) were extracted from the previous study reported by Gupta et al [65].
